# BoostedEnML: Efficient Technique for Detecting Cyberattacks in IoT Systems Using Boosted Ensemble Machine Learning

**DOI:** 10.3390/s22197409

**Published:** 2022-09-29

**Authors:** Ogobuchi Daniel Okey, Siti Sarah Maidin, Pablo Adasme, Renata Lopes Rosa, Muhammad Saadi, Dick Carrillo Melgarejo, Demóstenes Zegarra Rodríguez

**Affiliations:** 1 Department of Systems Engineering and Automation, Federal University of Lavras, Lavras 37203-202, MG, Brazil; ogobuchi.okey@estudante.ufla.br; 2 Faculty of Data Science and Information Technology (FDSIT), INTI International University, Nilai 71800, Malaysia; 3 Department of Electrical Engineering, University of Santiago de Chile, Santiago 9170124, Chile; pablo.adasme@usach.cl; 4 Department of Computer Science, Federal University of Lavras, Lavras 37200-000, MG, Brazil; renata.rosa@ufla.br (R.L.R.); demostenes.zegarra@ufla.br (D.Z.R.); 5 Department of Electrical Engineering, University of Central Punjab, Lahore 54000, Pakistan; muhammad.saadi@ucp.edu.pk; 6 Department of Electrical Engineering, School of Energy Systems, Lappeenranta-Lahti University of Technology, FI-53851 Lappeenranta, Finland; dick.carrillo.melgarejo@lut.fi

**Keywords:** Internet of Things, ensemble algorithms, cyberattacks, machine learning IDS, data imbalance, SMOTE, BoostedEnML

## Abstract

Following the recent advances in wireless communication leading to increased Internet of Things (IoT) systems, many security threats are currently ravaging IoT systems, causing harm to information. Considering the vast application areas of IoT systems, ensuring that cyberattacks are holistically detected to avoid harm is paramount. Machine learning (ML) algorithms have demonstrated high capacity in helping to mitigate attacks on IoT devices and other edge systems with reasonable accuracy. However, the dynamics of operation of intruders in IoT networks require more improved IDS models capable of detecting multiple attacks with a higher detection rate and lower computational resource requirement, which is one of the challenges of IoT systems. Many ensemble methods have been used with different ML classifiers, including decision trees and random forests, to propose IDS models for IoT environments. The boosting method is one of the approaches used to design an ensemble classifier. This paper proposes an efficient method for detecting cyberattacks and network intrusions based on boosted ML classifiers. Our proposed model is named BoostedEnML. First, we train six different ML classifiers (DT, RF, ET, LGBM, AD, and XGB) and obtain an ensemble using the stacking method and another with a majority voting approach. Two different datasets containing high-profile attacks, including distributed denial of service (DDoS), denial of service (DoS), botnets, infiltration, web attacks, heartbleed, portscan, and botnets, were used to train, evaluate, and test the IDS model. To ensure that we obtained a holistic and efficient model, we performed data balancing with synthetic minority oversampling technique (SMOTE) and adaptive synthetic (ADASYN) techniques; after that, we used stratified K-fold to split the data into training, validation, and testing sets. Based on the best two models, we construct our proposed BoostedEnsML model using LightGBM and XGBoost, as the combination of the two classifiers gives a lightweight yet efficient model, which is part of the target of this research. Experimental results show that BoostedEnsML outperformed existing ensemble models in terms of accuracy, precision, recall, F-score, and area under the curve (AUC), reaching 100% in each case on the selected datasets for multiclass classification.

## 1. Introduction

Monitoring computer networks in recent times has become more convenient and efficient through the use of intrusion detection systems (NIDS) that detect all abnormal actions on the network interface and report the same for proper actions. Concerning the growth in the size of communication networks, the application of NIDS and network intrusion prevention systems (NIPS) have undoubtedly become crucial in the 21st century network era. While the former (NIDS) detects intrusion and raises alarms to alert network experts to possible invasions in the network, the latter ensures that the alerting action does not harm the target system by preventing the attack. Both are implemented in synergy to ensure the holistic security of the network. Knowing when a system is under attack is paramount, as preventing the attack is essential. Hence, it accounts for researchers’ high interest in the intrusion detection system (IDS) domain. IDS monitors network traffic flow for possible cyberattacks and privacy violations at different layers of the network and prevents observed attacks from occurring.

The advance in technology has recently resulted in increased data collection sources in the IoT network ranging from smart devices, smart homes, and smart grids to  network devices such as routers, hubs, and switches, among others. Data collected from these end devices or network nodes are based mainly on the modes involved in monitoring the traffic. In IDS, there are three modes of monitoring network traffic flow: host-based [1,2], network-based, and hybrid [3] methods. When the IDS is implemented on the network nodes (host devices) to capture packets in transmission, filter the packets, and define whether they are real or malicious, the situation is known as host-based IDS (host-IDS). In some scenarios, the network administrator may decide to monitor the traffic at the network layers without including the end nodes of the network. Hence, the IDS is installed on the devices in the network layer or along the network transmission lines, and it is called a network-based IDS (network-IDS). In contrast to the above scenarios, the IDS is implemented by integrating the host-based and network-based methods to obtain a hybrid-IDS. Ideally, a network-IDS achieves a more comprehensive security advantage over other forms of IDS implementation modes because of a network-IDS interface with host systems and intermediate devices that allows it to prevent attacks on the lower interface at a quicker instance. Regarding network architecture, requirements, and specifications, multiple IDS systems or even hybrid can be used in a single network to track traffic in different end devices. IDs can also be categorized into misuse-based IDS, anomaly-based IDS, and hybrid, according to the nature of the network profile monitored by design. While the former works on the principle of known attacks, the latter is based on traffic flow patterns.

Internet of Things (IoT) systems are increasingly proliferating in every aspect of human existence, including finance, government, military, agriculture, and other industrial establishments. This increase has resulted due to the recent use of technologies such as Internet of Multimedia Things (IoMT), Industrial Internet of Things (IIoT), smart grids, smart and precision agriculture, Industry 4.0, and others [4]. Following this expansion in IoT application domains, a large volume of data is generated and transmitted over the Internet, resulting in more cybersecurity concerns to avert constant threats to IoT systems from individuals with malicious intentions. Many threat attempts, including DDoS, denial of service (DoS), web attacks, infiltration, and man-in-the-middle attacks, are some of the prevailing intrusive cyberattacks on IoT systems. In [5], the authors presented a comprehensive review of the different proposed models for IoT intrusion detection with ML classifiers, suggesting the high demand for highly efficient, effective, and accurate models developed with a machine and deep learning algorithms for IDS in IoT networks. An IDS deployed for an IoT system should be able to analyze data packets and produce real-time responses, obtain and critically evaluate data packets transmitted between multiple layers of the IoT network, and adapt to a variety of technologies in the IoT environment. This ideology serves as a principle for the development of IoT-based IDS models [6,7]. Operating in a constrained environment of low processing capabilities, dealing with fast response, and high-volume data processing should always be considered when designing IDS for IoT systems.

In the flow of events during IDS implementation, the IDS generates alerts when any suspicious activity is observed in the network. These alerts generated by the IDS at every entry point in the network are transmitted to the network monitoring expert (NME), which can either be a human or intelligent system, for analysis and consequently take possible actions. One of the current challenges with this scenario is the rate of false alarms generated by the IDS that may result in alert fatigue and failure in the system. In a case where there is a prevalence of alert fatigue, the network experts may spend unnecessary time investigating many false alarms and less time responding to realistic attacks. Hence, the need to reduce false-alarm rates has been studied in the literature [8,9]. In the case of a botnet attack that floods the entire IoT network with streams of bots causing resource depletion and network service interception, artificial intelligence (AI) devices are necessary to detect such floods.

The traditional method in monitoring network flows is the use of human experts who can easily become overwhelmed with false-alarm fatigue. Intelligent machine experts can overcome this problem. ML approaches to monitor both misuse and anomaly-based network traffic have been investigated with different performances in terms of accuracy, precision, recall, and F1-score. In [10], an expanded survey on the various implementation of ML in NIDS relating to IoT environment was presented.

According to [11], authors proposed an IDS based on ensemble ML. The system achieved an accuracy of 99.3% during testing. Other authors also achieved high degrees of accuracy in their proposals [12,13,14]. One major problem in the domain of IDS models using ML has been the rate of false alarms which continues to reduce the practicality rate of implementation of IDS. When systems are designed for the purpose of recommendation activities or for filtering emails into spam or not, the impact of false negatives (FNs) and false positives (FPs) may be neglected [15]. However, when it concerns intrusion whose effect can be more disastrous, reducing the FPs and FNs to their most feasible minimum is extremely important.

In this paper, we propose an IDS that uses boosted ensemble ML classifiers (BoostedEnML) aimed at enhancing the performance of IDS models in attack detection and classification with reduced false-alarm rates. Network packets are processed using ML algorithms to detect, analyze, and classify the traffic into their respective categories so that triggered alerts can be more reliable, reducing the computational overhead cost of managing false signals in the system. We implement our proposed model based on boosting algorithms as they showed better performance over other algorithms tested in this paper in model complexity, accuracy, and time function. Furthermore, the use of BoostedEnML in this work demonstrates that boosting classifiers such as LGBM and XGB can be combined to significantly improve the detection rate of ML IDS models in classifying attacks in an IoT environment as opposed to existing ML IDS models, which did not implement the combination of these two algorithms.

In the proposed IDS, we train, validate, and test different models based on random forest (RF) [16], AdaBoost [17], XGBoost [18], LightGBM [19], extra tree (ET), and decision tree (DT) [20] classifiers. Except for the DT, other algorithms already exist as ensemble classifiers based on the aggregation of various DT algorithms. A combination of these using a new method usually results in improved performance, in our case, for research. We develop the proposed model on the CSE-CIC-IDS2018 and CIC-IDS2017 datasets, which are the most comprehensive datasets for IDS development [21] currently available. IDS models and, generally, ML algorithms generalize better on balanced data by learning the same features from each class in the dataset. The two datasets used for this work contain imbalance; therefore, we handle the imbalance in our dataset using two main oversampling techniques, which are synthetic minority oversampling technique (SMOTE) [22] and adaptive synthetic sampling (ADASYN) [23,24]. In the NIDS domain, several ensemble ML approaches have been discussed [25,26,27,28,29,30,31], but none have used these classifier combinations to the best of our knowledge. Our proposed approach detects intrusion more accurately and precisely compared to existing systems [25,26,27,29].

The key contribution of this research are outlined as follows:

1. A search algorithm based on GridSearchCV was implemented to select the most fundamental parameters necessary to obtain a high-performing IDS model. This ensures that the model learns holistically on the dataset.
2. We performed feature selection to obtain the most predominant features of the datasets and used an ensemble technique to combine the features to obtain a comprehensive array of best performing features.
3. We implemented oversampling techniques, such as SMOTE and ADASYN, to handle data imbalance in our two datasets, thereby obtaining a highly accurate classification model. These datasets are widely used in similar and recent research.
4. We implemented several ensemble models and selected the best models depending on time-cost function and overall accuracy. Models based on boosting algorithm showed better performance; hence, they were used to develop the BoostedEnML as proposed. In each step, the resulting model was validated for a multiclass classification task.
5. We evaluated the model performance on two robust datasets having various intrusion attempts and used the AUC to validate the performance accuracy.

On evaluation, experimental results show that the proposed BoostedEnML IDS model accurately classified the network traffic flows in the used datasets with reduced FN, FP, and FAR, and maintained a high detection rate for packets of data on the IoT network. Our IDS model for IoT systems showed improved performance over existing models discussed in the literature. In addition, the proposed approach helps to reduce the model complexity by using lightweight algorithms to develop the ensemble model. With the grid search cross-validation applied, we ensured that the proposed model learns from the most relevant network traffic features and uses the algorithm’s best parameters to save training time.

The rest of the paper is organized in the following pattern. Section 2 presents the background of ML in IDs, selected algorithms, and related propose works. Our approach to achieving the proposed model is presented in Section 3. In Section 4, we present, analyze, and interpret our research findings, and then we conclude our paper in Section 5.

## 2. Background and Related Work

Currently, many research breakthroughs exist in the IDS for network security applied to IoT systems. Notwithstanding, there still exist significant challenges, some of which include a lack of a consistent understanding of normality introduced by network unpredictability, heterogeneous nature of network traffic, unavailability of appropriate public IDS datasets, and vulnerable environments and loopholes that grant access to attackers who actively search for and exploit security flaws. Some security researchers have opined that these challenges are uniquely inherent in IDS in networks and may not be observed in other domains [32]. IoT system security challenges are evolving with the expansion of the application domain of the technology. The IoT layers comprising the perception, the network, and application layers continuously face different threats. The application layer sitting at the topmost part of the network transmits information between the network and other services and tends to face most of the threats due to the connection interface established between other devices [5]. In [33], authors proposed an ensemble IDS model for the IoT environment using gradient boosting algorithm for a binary class classification task. The proposed model reached an accuracy of 98.27% and a precision of 96.40% using XGBoost for feature selection.

Data generation in IoT systems has witnessed a great expansion in the last decades, and transmitting such a volume of data over a regular network has been challenged with high computational resource requirements, low bandwidth, and advanced network attacks. One approach to overcoming the resource constraint and increased cyberattacks is using a cloud computing environment with massive storage capacity, high computational power, and configurable resources integrated with virtualization capabilities for data storage [34]. Flooding the IoT network at all layers with DDoS attacks such as UDP flood, ICMP/Ping flood, SYN flood, ping of death, and zero-day DDoS attacks have resulted in high data loss. Nie et al. in [35] proposed a novel intrusion detection system in the IoT domain to deal with such intrusive attacks as distributed denial of service (DDoS), packet-sniffing, and man-in-the-middle attacks. The authors used the GAN method to train an IDS model using the CSE-CICIDS2018 and CICDDoS2019 datasets, the most recent and complete datasets for training and testing IDSs. The research showed that the models achieved about 97% accuracy in both datasets in the training and evaluation phases. Mitigating DDoS, DoS, botnet, and infiltration attacks on the IoT networks has recently been a challenging task [36].

In [37], authors proposed many IDS models based on machine learning to mitigate attacks on IoT devices in the smart city setting. Different ML algorithms and ensemble methods, such as the stacking, bagging, and boosting methods, were used to develop the ensemble model. On evaluation, the proposed ensemble models reached an accuracy and recall of 0.999. Several Ml algorithms were used by [38] to propose the IDS model for IoT networks. In the work, the authors used K-nearest neighbor (KNN), support vector machine (SVM), artificial neural network (ANN), and other ML algorithms in their work. The models were trained using the train–test split method at an 80:20 ratio; the resulting models were evaluated on the BoT-IoT dataset and achieved an accuracy of 99% with the KNN. Furthermore, Ref. [39] proposed an IDS model for cyberattack monitoring based on the bagging ensemble method with an accuracy of 99.67% on the NSL-KDD dataset.

Currently, several open-source network monitoring solutions are leveraged to provide network security by capturing the TCP/IP packets in the networks. Suricata [40] and Snort [41] are the most commonly used open-source traffic monitoring software. Both have shown some limitations in recording attacks during operation. Suricata and Snort work based on predefined rules to detect malicious attacks [42,43]. One of the major drawbacks of these systems is that any deviation from the predetermined rules would result in a false alarm. Again, it requires that a security expert study both existing attacks and novel network deviations under defined conditions that define the database’s signatures. Attackers exploit the vulnerabilities regularly discovered in IoT networks and use the same to tamper with the events protocol. Since this process is dynamic, using a manual approach to define attack features can be ineffective and burdensome to handle.

In addition, considering the extensive data generated by the IoT systems, manually searching for attacks in the dataset can be a hassle. An attempt to proffer a solution is the application of machine learning, which today has gained exceptional popularity in industries and the scientific community in IoT cybersecurity [44,45,46,47,48]. The machine learning technique primarily used in IDS systems is supervised learning, where the database is provided with features and labels to classify the network traffic. Ensemble learning defines an approach where several base learners, referred to as weak learners, are aggregated based on specific rules to form a stronger classifier algorithm [49]. With ensemble methods, models achieve better performance in predicting the nature of the traffic flow, as overfitting and class imbalance are handled with a better approach [50]. In a nutshell, many of the existing ensemble models implement DT architecture in a bagged or boosted manner, leading to improved results.

The bagging method uses different samples of the train data on the algorithms at different times and rates, resulting in different submodels whose average is the desired output of the training. The voting ensemble uses majority voting (soft or hard) for classification tasks, as used in [51] with an accuracy of 99%, and averaging for regression tasks to combine the outputs of the base learners. Bagged DT and RF models are the most widely used bagging ensemble models [50]. On the contrary, the boosting algorithm forces each weak classifier to concentrate on a specific component of the data in the training distribution, thereby transforming groups of weak classifiers into strong ones with improved accuracy. Through this approach, later learners are pressed to concentrate on the mistakes made by earlier learners. Hence, the later classifiers are trained to overcome the mistakes of the earlier classifiers. As a result, each baseline learner in the boosting ensemble can concentrate more on the data points that the other learners misunderstood. When the data are pooled, boosting produces a more precise prediction [17].

### 2.1. Machine Learning Models

In this section, an overview of the selected ML algorithms used in this work is presented. For simplicity, we discuss the decision tree, AdaBoost, extra tree, random forest, LightGBM and XGBoost.

1. Decision Tree (DT): Decision trees (DTs) are data structures composed of elements called nodes. Following a hierarchical model, the tree has a root node, where the tree begins; sequentially, the tree is composed of child nodes, where each node can have other children or subtrees. A leaf or terminal node is a node that has no offspring. The initial data enters the tree’s root and passes through the decision nodes until reaching the leaf node, which presents the result of the processing. Usually, three main variations of DT are prominent in use for IDS designs: ID3 [52], C4.5 [53], and CART [54].
2. Adaptive Gradient Boosting (AdaBoost): Freund et al. [17] proposed the AdaBoost as a boosting learner that creates a chain of classifiers in succession on the same dataset in such a manner that subsequent classification improves on the errors of the earlier classification. The algorithm achieves this by assigning higher weights to the incorrectly classified classes and lower weights to the correctly classified classes, thereby ensuring that the incorrectly classified instances gain priority during the next phase. The exact process repeats until the best possible result is achieved and the algorithm has used all the instances in the data. As implemented in [55], authors proposed an IDS based on AdaBoost using the CIC-IDS2017 dataset as a training dataset. Applying SMOTE, an accuracy of 81.31% and an F-score of 81.31% were achieved during testing. Although achieving good accuracy, this resulted in a lot of false predictions that need to be improved.
3. Extra Tree (ET) Classifier: This algorithm improves the performance of DT and RF by incorporating a more significant number of trees into its network. As a result, compared with other ML algorithms, it has the highest number of trees and computational resource requirements. This algorithm works on the principle of meta-estimator and applies an averaging rule to increase predicted accuracy and reduce overfitting. First, the meta-estimator fits several randomized decision trees on different subsamples of the same dataset. Then, it aggregates the results of multiple decorrelated decision trees collected in a forest to output a classification result. The package is available in the *sklearn.ensemble.ExtraTreesClassifier* library for use in any ML tasks [56].
4. Random Forest: This algorithm, proposed by Breiman [16], has shown great results in both classification and regression problems, making it the most used ensemble algorithm. By constructing component trees, the algorithm reduces the connection of different decision trees. It extends the attributes of bagged decision trees by inculcating randomized attributes. More importantly, the performance gains observed in RF are achieved through the randomness in the attribute selection process, not from the splits in the decision trees which are created based on a subset of the data attributes [15]. As a popular ensemble algorithm, several authors have used it in IDS [57,58,59]. In [58], authors proposed an IDS model which used principal component analysis (PCA) for dimensionality reduction and random forest classifier for classification. The result was compared with support vector machines (SVM), naive Bayes, and classical decision trees. On testing, authors claimed that the model achieved an accuracy of 96.78%, making it preferable over the others, which achieved less accuracy.
5. Extreme Gradient Boosting (XGBoost): Extreme gradient boosting (XGBoost) [60] is an extension of the implementation of gradient boosting tree proposed by Friedman et al. [61]. Because it offers parallel computation, cache awareness, a built-in regularization strategy to avoid overfitting, and tree optimization by a split-finding algorithm, XGBoost generally outperforms gradient boosting in terms of performance as it has a quick training and inference time. In [62], an efficient IDS model based on XGBoost was proposed for computer networks. The model was trained and evaluated on the network socket layer–knowledge discovery in databases (NSL-KDD) dataset with an accuracy of 98.70%.
6. Light Gradient Boosting Machine (LightGBM): Observing the high training time requirement for gradient boosting decision trees (GBDT), Ke et al. [19] proposed two novel techniques to overcome the challenge based on Gradient-based One-Side Sampling (GOSS) and Exclusive Feature Bundling (EFB). This new implementation was named LightGBM, and it improved training and inference time of GBDT by 20%. Since its development, it has shown highly impressive results even in IDS systems, as shown in [63,64].

### 2.2. SMOTE and ADASYN for Imbalanced Dataset

One of the many challenges affecting the efficiency of ML models is the inadequacy of data points in the dataset used to train the models. Hence, the model cannot learn comprehensively from the available data, creating room for incomplete knowledge in some instances. In the case of our dataset, there are over 13 million benign traffic in the CSE-CIC-IDS2018, with some attacks such as SQL injection having only 87 data instances. In addition, in the CIC-IDS2017, the heartbleed attack has only 11 instances compared with the benign instances with 2 million data points. Some techniques have been proposed to solve this problem, usually based on either oversampling or undersampling methods. In undersampling, the majority class is reduced to be suitable to the minority classes, which leads to the loss of vital information, while in oversampling, the minority classes are increased to be equal or approximate to the majority classes. SMOTE [22] and ADASYN [23] are two of the many oversampling techniques used in handling data imbalance. SMOTE first selects a minority class instance *r* randomly and finds its K-nearest minority class neighbors. The synthetic instance is then created by choosing one of the K-nearest neighbors *p* at random and connecting *r* and *p* to form a line segment in the feature space. Finally, the synthetic instances are generated as a convex combination of the two chosen instances, *r* and *p*. ADASYN is based on the idea of adaptively generating minority data samples according to their distributions: more synthetic data are generated for minority class samples that are harder to learn compared to those minority samples that are easier to learn. Other derivatives of the SMOTE method include borderline-SMOTE [65], borderline-SMOTE SVM, SMOTEN, SMOTENC, and KmeansSMOTE, which are all available in the Imblearn-learn library [66].

### 2.3. Ensemble Machine Learning

Ensemble learning in ML aggregates the results of different ML classifications aimed at achieving better performances in accuracy and attack classification detection rate. In ensemble learning (EL), homogeneous and sometimes heterogeneous algorithmic classifiers can be combined to build an improved predictive model with better inference time [67]. The applicability of ML techniques differs between use cases and the characteristics of the dataset on which it is built. This implies that the technique used for one project dataset might not be applicable to another of the same or similar domain [68]. Hence, EL tries to achieve a model that can be used in the application domain with better results. Different EL models perform differently from each other in the IDS domain based on the dataset used to develop the model. Usually, three main classes/methods of EL exist, including bagging, stacking, and boosting.

Bagging entails averaging the predictions from many decision trees that have been fitted to various samples of the same dataset. It usually incorporates three main approaches, including bootstrapping samples of the train dataset, fitting unpruned DTs on each sample, and use of simple voting or averaging of predictions to obtain the final results. Some known examples of this include bagged decision tree (BDT), random forest (RF), and extra tree (ET) [69]. Given a training set T=t1,…,tn with responses L=l1,…,ln , the bagging algorithm repeatedly (P times) selects a random sample accompanied by replacement of the training set, then fits trees of different sizes to these samples. This can be achieved using the procedure shown in Algorithm 1.

(1) $$\hat{f}=\frac{1}{P}\sum_{b=1}^{P}f_{b}\left(x^{\prime }\right)$$

**Algorithm 1** The algorithm for bagging classifier
1:**for** b=1,…P: **do**2:     Sample, with replacement, *n* training examples from T, L; call these Tb, Lb.3:     Train a classification tree, fb on Tb, Lb.4:     After training, predictions for unseen samples *x*′5:     obtain the final predictions from all the individual fb on *x*′ by taking the average of all predictions for regression or taking the majority vote for a classification problem using Equation (1).6:**end for**


This approach leads to a better model with reduced variance of the IDS model without increasing the bias. This shows that in a case where the predictions of a single tree are extremely noise-sensitive on the training set, as long as the trees are not correlated, the average of the trees is insensitive to noise. Hence, bagging yields reliable IDS models for IoT environment. When we train many trees on a single dataset (training data), the trees would produce strongly correlated trees (even with the same tree many times not considering whether the training algorithm is deterministic or nondeterministic), which tends to cause overfitting and bias; bagging or bootstrapping the samples in the datasets is a measure to ensuring decorrelation in the trees by showing them different samples of data during the training process in the training sample [70]. More specifically, we calculate an estimate of the uncertainty of the prediction as the standard deviation, σ of the predictions from all the individual regression or classification trees on x′ according to Equation (2):

(2) $$\sigma =\sqrt{\frac{\sum_{b=1}^{P}{\left(f_{b}\left(x^{\prime }\right)-\hat{f}\right)}^{2}}{P-1}}$$

Stacking, also known as stacked generalization, is an ensemble modeling technique that includes using data from many models’ predictions as features to construct a new model and make predictions. In other words, during stacking, we fit different models on the same train data, obtain the results of the predictions, and use another algorithm to combine the predictions for improved results. This approach ensures that the learned features from the first model are maintained by the second model, thereby showing improved results compared to the single model. By using heterogeneous weak models trained on the same data sample, more robust IDS models are obtained [71,72]. Popular EL algorithms based on stacking are blending and super ensemble.

When boosting is implemented, there is sequential addition of the members of the ensemble algorithms which corrects the predictions of the previous classifier and generates a weighted average of the predictions as the output. This feature of boosting algorithm accounts for their better performances over stacked and bagged ensemble classifier. Common examples include AdaBoost, XGB, LGBM, and GBDT [50,73]. Assuming that the boosting ensemble is defined in terms of weighted sum of *L* weak learners, we obtain the function shown in Equation (3) where $c_{l}$ are coefficients and $w_{l}$ are weak learners.

(3) $$s_{L}\left(.\right)=\sum_{l=1}^{L}c_{l}Xw_{l}\left(.\right)$$

One drawback of this approach is the difficulty to achieve faster optimization convergence. To arrest this challenge, instead of solving for the coefficients and the weak learners in one try, we implement an iterative optimization approach that is more cost-efficient and tractable. In this scenario, each weak learner is added one by one, checking the iteration for the best possible pair that it gives (coefficient and weak learner) to update the current ensemble model. Hence, we define recurrently the value of $s_{l}$ in a way such that

(4) $$s_{l}\left(.\right)=s_{l-1}+c_{l}w_{l}\left(.\right)$$

In which case the values of $c_{l}$ and $w_{l}$ are selected such that $s_{l}$ is the model which has the best fit on the train data, therefore it presents the best possible improvement over $s_{\left(l-1\right)}$ according to Equation (4). If we define E(.) as fitting error of the given model and e(.,.) to be the loss/error function, we denote the following:

(5) $$\left(c_{l},w_{l}\left(.\right)\right)=\underset{c,w(.)}{argmin}E\left(s_{l-1}\left(.\right)+cw\left(.\right)\right)=\underset{c,w(.)}{argmin}\sum_{n=1}^{N}e\left(y_{n},s_{l-1}\left(x_{n}\right)+cw\left(x_{n}\right)\right)$$

As a result, rather than optimizing “globally” over all of the *L* models in the total, we approach the optimum by optimizing “locally” creating and gradually adding the learning algorithm to the strong model. Hence, Equation (5) presents a comprehensive approach to the design of highly optimized ensemble classifier based on booting technique. A typical algorithmic representation of the procedure for implementing the boosting algorithm is shown in Algorithm 2 with primary focus on the AdaBoost classifier upon which other boosting classifiers are built. A summary of related literature reviewed in this section is presented in Table 1.
**Algorithm 2** The algorithm for boosting classifier
1:Form a large set of sample features2:Initialize the weights of training samples3:**for** T rounds **do**:4:     Normalize the weights of the samples5:     For available features from the set, train a classifier using a single feature and evaluate the training error6:     Choose the classifier with the lowest error7:     Update the weights of the training samples: increase if classified wrongly by this classifier, decrease if correctly8:**end for**9:Form the final strong classifier as the linear combination of the *T* classifiers.


## 3. Materials and Methods

**Figure 1:**
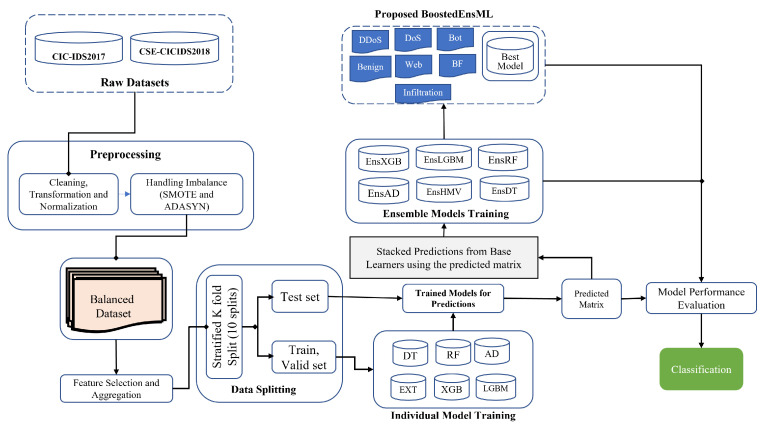
Design architecture of BoostedEnML for IDS in IoT systems.

The materials used for this research and the method are discussed in detail in this section. The well-elaborated architecture describing the process flow is given in Figure 1. The methodology is specifically divided into five different phases, namely, (a) data collection, (b) data preprocessing, (c) ensemble feature selection, (d) model classification, and (e) anomaly detection (classification). We begin the proposed IDS model design by checking the database for important datasets that best meet the specific objectives of this paper. Data in raw format are composed of irregularities and misinformation that must be preprocessed. We perform feature engineering to remove redundant features, then develop the IDS model, as shown in Figure 1. In the end, the final model proposed in this work is implemented using the pseudocode presented in Algorithm 3.

### 3.1. Data Collection

The performance of the ML model is as important as the data used in the training process. For this reason, in our work, we searched through the available datasets to select the most wide and comprehensive datasets upon which we could build our IDS model. Two recent datasets were selected which are publicly available for research purposes: CICIDS2017 (http://205.174.165.80/CICDataset/CIC-IDS-2017/Dataset/, accessed on 7 February 2022), consisting of over 2 million instances among which 83% are benign and 17% are attack classes, and CSE-CIC-IDS2018 (https://registry.opendata.aws/cse-cic-ids2018/, accessed on 7 February 2022), comprising 83% benign and 17% attack, were used in this paper. The datasets collected from these sources are contained in different folders in CSV format. To obtain a robust dataset, we first aggregated all the different CSV files into a single file for each of the selected datasets. The data contain relevant information of the problem domain and needed to be cleaned for further analysis. The selected datasets are maintained by the Canadian Institute of Cybersecurity and the University of New South Wales [76]. Other commonly used datasets include KDD Cup’99, NSL-KDD, UNSW-NB15, Bot-IoT, CICDDoS2019. Usually, the dataset is divided into train and test portions. The collected dataset contains information about network flows recorded in forward and backward order. Some of the features of the datasets include source IP, destination IP, timestamp, flow duration, flow bytes, etc.
**Algorithm 3** The algorithm for the BoostedEnsML
1:Define the number of folds in the split, s2:Initialize s = 03:**while** s ⩽ 10 **do**:4:     Train the selected classifier (LGBM or XGB) using 9 parts of the 10 folds and perform prediction on the other part5:     XGB and LGBM are used for predictions on the train set and test data.6:     s += 17:**end while**8:Using Stacking Classifier to combine the predictions from the two base models.9:BoostedEnsML is applied to the test data to make final predictions.


### 3.2. Data Preprocessing

The raw datasets shown in Table 2 and Table 3 consist of 15 different classes each and one benign class. There are different web, DDoS, and DoS attacks in the datasets. We merged these related attacks into their respective classes. For instance, the DoS GoldenEye, DoS slowloris, and DoS Slowhttptest in the CSE-CIC-IDS2018 dataset were merged into the DoS attack, while in the CICIDS2017, the DoS Hulk, DoS SlowHTTPTest, DoS GoldenEye, and DoS Slowloris were also merged. Similarly, the same approach was used to merge the DDoS flows in both datasets. Usually, data come in raw form and cannot be implemented in that form in ML algorithms. It is important that the crude datasets are cleaned, sanitized, transformed, and features reduced to ensure that attack features used in the ML classifier are the best features. In cleaning and sanitizing the datasets, we removed duplicate rows and columns; rows containing special characters (@,#,%) were checked, and such special characters were deleted. We noticed that some instances in the dataset were ’inf’ and NULL values, so the null value rows and columns were deleted. This cleaning was performed on both datasets. ML classifiers can correctly handle all numeric inputs; we converted all non-numeric data into numeric using the LabelEncoder. LabelEncoder is used in ML to encode the *y*- label into numeric values in the range of 0 to n_classes -1.

For better understanding of the correlation between the traffic features in the dataset, we performed statistical analysis including univariate, bivariate, and multivariate analysis using data visualization tools such as Matplotlib, Seaborn, and Plotly. We observed in the dataset during exploratory data analysis (EDA) that most of the numeric data are of higher values than others. For this reason, we used MaxAbsScaler (Maximum Absolute Scaler) to transform the data into the range of zero and one (0 and 1). There exist the standard scaler, min max scaler, and robust scaler. One advantage of the Max Absolute Scaler over other feature transformation techniques is that it estimates, scales, and transforms each feature one by one in such a way that the maximum value of each feature in the train dataset will be 1.0; hence, the center of the data is maintained and sparsity is not destroyed.

As shown in Table 2 and Table 3, our datasets contain severe data imbalances, having benign features in millions and attacks in thousands and hundreds. To handle the imbalance, we used two different sampling techniques to reduce the dataset size without affecting the model’s performance. We first oversampled the minority classes using SMOTE and ADASYN. These data sampling techniques generate synthetic instances for the minority class using its features. They ensure that the original information contained in the dataset is maintained. Next, we reduced the benign class using a random undersampling technique, which randomly removes some samples of the benign class without affecting its contribution to the model performance. Similar attack types were merged to obtain seven labels for CSE-CICIDS2018 (consisting of six attacks and one benign feature) and nine for CIC-IDS2017 (consisting of eight attacks and one benign feature).

Next, we needed the training, validation, and test datasets. The Sklearn library provides the train–test split function for splitting the dataset, while the Keras module provides the train-test–validation split option. In this research, we used the *StratifiedKFold* cross-validation split function to achieve better performance. StratifiedKFold was used to split the data into ten different subsets or folds, and in each training iteration, nine different subsets were used for the training and validation, while one was used for testing the performance of the model. The process was repeated ten times until all the samples in the folds were used, thus ensuring that each data point participated in the model training. By using this method, data leaking, which occurs when some test data are visible during training and causes the model to be biased toward the test data, may be avoided.

### 3.3. Hyperparameter Optimization and Ensemble Feature Selection

Hyperparameter optimization (HPO) is an automated method for picking classifier parameters to train the model. While model parameters (MPs) are learned and updated by the model during training, ML programmers define the hyperparameters for the classifier. This paper employed two search strategies to find the optimal hyperparameters for improving model performance. The RomandomizedSearchCV and GridSearchCV were used. While the RandomSearch algorithm randomly selects parameters based on the search space provided to each holding other parameters constant, the GridSearch CV exhaustively searches the grid of parameters and reports the best candidate parameters. Depending on the number of iterations (n_iter) defined, the RandomSearch can be faster than the GridSearch. Usually, the parameters to be tuned are defined based on the ML algorithm being implemented. For instance, in RF, the parameters tuned are max_features, n_estimators and oob_score, whereas the max_depth, n_estimators and learning_rate are tuned for the XGBoost classifier. When we compared the tuning results, we discovered that the parameters produced with GridSearchCV improved the model’s performance more than those obtained with RandomSearchCV; thus, all future training of all models was based on the GridSearchCV-tuned parameters. As previously indicated, some variables in the dataset are unimportant because they have little influence on the traffic flow characteristics. Hence, we used feature selection to determine which features contribute the most to determining network flow characteristics. Therefore, the random forest algorithm for feature importance was implemented, and the first 64 most important features, evaluated by the RF feature importance method, were chosen. Thus, our models were developed based on the chosen 64 features. Reducing these features helps to lower the model complexity and improve training cost while achieving the same performance output. The selected feature map is shown in Table 4.

### 3.4. Model Selection and Training

In IDS implementation, detecting various forms of network intrusion requires IDS to be capable of functioning in multiclass mode. Hence, our task is a multiclass task. ML algorithms are widely used in this domain [77,78]. In this paper, decision tree (DT), extra tree (ET), random forest (RF), AdaBoost (AD), XGBoost (XGB), and LightGBM (LGBM) are the selected algorithms. In research on boosted algorithms, DT, ET, and RF were used as a baseline to evaluate the computational complexity of the boosted algorithms and the resulting BoostedEnML. First, six different models were developed individually for each of the algorithms; and their performances were evaluated. In the ensemble model design, each of the classifiers, DT, RF, XGB, LGBM, were aggregated. Then, to obtain an ensemble IDS model based on DT, other models were used as estimators while DT was used as the meta-learner. This approach was repeated for all the classifiers to obtain the desired results: ensemble decision tree (Ens_DT), ensemble random forest (Ens_RF), ensemble AdaBoost (Ens_AD), ensemble XGBoost (Ens_XGB), ensemble LightGBM (Ens_LGBM). We compared the different ensemble methods by implementing an ensemble using voting and stacking classifiers. These two are called the ensemble hard majority voting (Ens_HMV) and ensemble stacking model (EnSM). Finally, we used the two boosting classifiers (XGB and LGBM) to develop the BoostedEnML which is proposed in this paper. The algorithm presented in Algorithm 3 helps in implemented the BoostedEnML IDS model. We began by defining and initializing the number of splits we wanted each of the folds to have. We used 10 K-folds in each split during which training was performed on 9 folds; the remaining 1 fold was used to validate the model performance. This process was repeated until the 10 folds were completed, thereby using all the data in the train set. Although other approaches show similar or related performances, we demonstrated that BoostedEnML can be used to achieve network traffic classification with high accuracy and reduced computational cost.

### 3.5. Evaluation Metrics

The metrics accuracy, precision, recall, F-score, area under the curve (AUC), confusion matrix, and receiver operating curve (ROC) were used to check how the model performed on the test data. In the field of ML, these metrics are highly used in evaluating the performances of trained models. While the accuracy is a very good evaluation metric for ML tasks, it is not highly recommended for multiclass classification tasks involving imbalanced datasets. This is because high accuracy on imbalanced data may not have resulted from a generalized learning attribute of the model. Hence, other metrics were combined in this work. Given that TP_os, TN_eg, FP_os, and FN_eg are the definition for true positive, true negative, false positive, and false negative outcomes of the models, respectively, the evaluation metrics can be defined by Equations (6)–(10) for the weighted macro performance of the model in terms of the accuracy, precision, recall, and F-score. TP_os represents the samples in our dataset that were correctly classified as positive, TN_eg are samples that were correctly identified as negative, FP_os represents the instances that were negative but were mistakenly identified as positive by the model, and FN_eg represents the positive instances that were classified as negative by the model. The confusion matrix shows the model’s performance in classifying each sample correctly or wrongly on a graph. The AUC–ROC curve was originally designed for binary problems; however, it can be adapted for multiclass problems using the OneVersesRest (OVR) or OneVerseOne (OVO) and ’multiclass’ arguments. The one-vs.-one algorithm is used to calculate the average of the ROC–AUC scores in pairs, and the one-vs.-rest algorithm calculates the average scores of the ROC–AUC for each network flow label against all other class labels, as shown in Equation (11). We can set the multiclass keyword argument in the function to ’ovo or OVR’ while the average is set to ’macro’. This way, we can use the AUC–ROC curve function for multiclass problems.

(6) $$Accuracy_{macro}=\frac{TP\_os+TN\_eg}{TP\_os+TN\_eg+FP\_os+FN\_eg}$$

(7) $$Precision_{macro}=\frac{TP\_os}{TP\_os+FP\_eg}$$

(8) $$Recall_{macro}=\frac{TP\_os}{TP\_os+FN\_eg}$$

(9) $${F-measure}_{macro}=\frac{2\times \left(precision\times recall\right)}{precision+recall}$$

(10) $$FPR=\frac{FP\_os}{FP\_os+FN\_eg}$$

(11) $$AUC=\frac{1}{c(c-1)}\sum_{j=1}^{c}\sum_{k>j}^{c}\left(\mathrm{AUC}\left(j|k\right)+\mathrm{AUC}\left(k|j\right)\right)$$

where *c* is the total number of classes and AUC(j∣k) is the AUC with class j as the positive class and class k as the negative class. In general, AUC(j∣k) ≠ AUC(k∣j) in the multiclass case [79].

Equation (12) extends Equation (11) for weighted ROC–AUC curves. The modification is to change the value for the average to *’weighted’* and other arguments are retained. The *’weighted’* [80] returns the prevalent weighted average for each of the class in the dataset.

(12) $$AUC=\frac{1}{c(c-1)}\sum_{j=1}^{c}\sum_{k>j}^{c}p\left(j\cup k\right)\left(\mathrm{AUC}\left(j|k\right)+\mathrm{AUC}\left(k|j\right)\right)$$

In the experimental setup for this task, we used Python Numpy, Pandas, Matplotlib, and the machine learning library Scikit-learn for the software. The code was executed on a computer running on Intel(R) Core(TM) i7-7700 CPU @ 3.60 GHz, 3600 Mhz, 4 Core(s), 16 GB (15.9 GB usable), Windows 10 Home Single Language 64-bit and NVIDIA GeForce GTX 1050 Ti GPU.

## 4. Results and Discussion

In this section, we present and discuss the results obtained from the experiment. As earlier stated, we performed the experiment using two well-known datasets: CIC-IDS2017 and CSE-CICIDS2018, which are publicly available for research purposes [76]. First, we oversampled the data points such that there were almost the same values for each of the samples. For instance, bot, which has 286,191 samples against the benign traffic, with 12,484,708 instances, in Table 2 needed to be increased, otherwise the model would only learn the features of the benign traffic since it would see more of the packets injected as benign. The datasets obtained for the training, validation, and testing after handling imbalances with SMOTE and ADASYN, and splitting using *StratifiedKfold* cross-validation, are presented in Table 5. For each of the nine class labels in CICIDS2017, there are 606,812 instances for training and 67,242 instances for testing. The same applied to the CSE-CIC-IDS2018 dataset.

**Figure 2:**
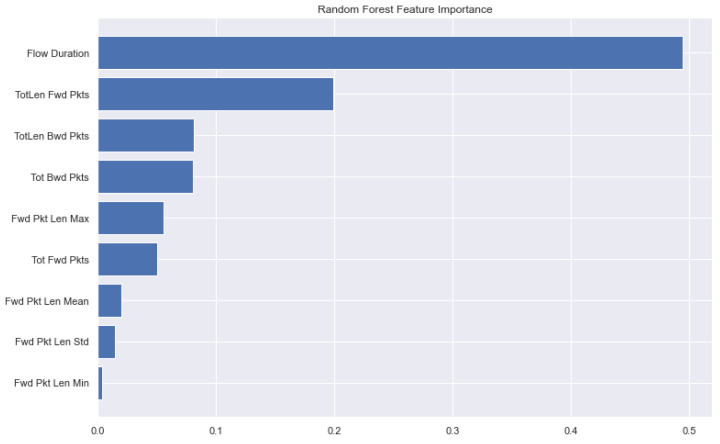
Feature importance extracted from the CICIDS2017 dataset using RFR.

After oversampling the datasets, the resulting data points were very high for the ML task; so we performed undersampling and selected a total of 5,189,072 (6%) data instances of the CSE-CICIDS2018, and 30% of the CIC-IDS2017 dataset with a total of 6,671,664 samples. The two datasets both had a total of 80 features each after preprocessing. A total of 64 features were selected, as shown in Table 4 with the Timestamp, Destination port, Fwd Seg Size, Min, and Init Fwd Win Bytes being the top four features in the CSE-CICIDS2018 dataset. The features are listed in ascending order with their importance according to weights attached to each. This helps us to understand the extent to which each feature is important to the model performance. Features such as ‘Tot Fwd Pkts’, ‘Subflow Fwd Pkts’, ‘PSH Flag Cnt’, ‘Idle Std’, ‘Idle Mean’, ‘Active Std’, ‘Active Mean’, ‘Active Max’, and ‘ACK Flag Cnt’ are observed to contribute almost one-thousandth (1/1000) to the model training and testing. This can imply that if these features are removed, the model can still perform very accurately. With the exception of the Timestamp and Destination Port (Dst Port), the most important features which contribute almost tens of percentages are the first four features: Fwd Seg Size, Min, Init Fwd Win Bytes, and TotLen Fwd Pkts. Figure 2 shows the first 10 important features in the CICIDS2017 dataset as generated with the random forest feature importance. First, we show the results obtained after training the models on the CSE-CICIDS2018 dataset. The performance of each model in terms of the accuracy, precision, recall, F-score, model size, and test time are presented in Table 6. The results show that the task classifies the labels into their respective seven classes as contained in the dataset; identifying, at each time, one of the categories of the network traffic. During the test, the accuracy for each of the ML algorithms, DT, RF, ET, AD, LGBM, and XGB, are 98.7%, 98.4%, 98.3%, 97.8%, 98.8%, and 98.9%, respectively. It can also be observed that XGB has the highest accuracy, precision, recall, F-score, and AUC, compared with other ML algorithms. Hence, it achieves the best performance in correctly identifying each network traffic according to its category. This is expected as it has shown very high accuracy in previous works, outperforming some deep learning models in some datasets [81].

Furthermore, the LGBM model follows the XGB having obtained accuracy, precision, recall, F-score and AUC of 98.8%, 98.83%, 98.83%, 98.83%, and 99.96%, respectively. LGBM is a lightweight version of the XGB algorithm specifically designed for timing optimization with high accuracy, as seen in this current task. In general, a close look at the evaluation metrics shows close, and almost the same, values obtained for each of the models for each metric used. For instance, DT achieved almost 99% for all the metrics, and RF achieved approximately 98% for all the metrics as well as ET classifier. This is achieved as a result of the balanced dataset and cross-validation approach used. In all cases, each algorithm generalizes very well on the traffic, and thereby gains knowledge to identify to which class the packet belongs. Since all the models trained on the algorithms have almost similar performances, we measured the train and test time for each model to enable us to select the most suitable model for further tasks of ensemble design.

As shown in Table 6, ET required the highest amount of time to predict the different attack classes, using about 15.1 s. This is attributed to the large number of trees in its architecture, so ET was excluded from being used as a base learner in ensemble models. DT, RF, LGBM, and XGB had total test times of 0.25 s, 9.98 s, 3.4 s, and 4.25 s, respectively. Therefore, we chose them as base learners for ensemble models.

The results obtained for the CIC-IDS2017 dataset using the various metrics are shown in Table 7. On this dataset, the DT, RF, ET, AD, LGBM, and XGB classifiers detected each class with an accuracy of 99.59%, 99.45%, 99.68%, 69.67%, 99.16%, and 99.51%, respectively. In terms of the AUC score for each of the classifiers, the DT, RF, ET, AD, LGBM, and XGB reached 99.76%, 99.98%, 99.97%, 67.9%, 96.81%, and 99.97%, respectively, with ET and XGB having the same AUC score of 99.97%. Considering the precision and recall performances of the six models, we observe that each model has high values, which demonstrates the capacity of each of them to give reliable predictions while detecting network traffic. In precision, the DT, RF, AD, LGBM, and LGBM classifiers reached precisely 99.59%, 99.48%, 99.68%, 66.76%, 96.96%, and 99.52%. These performances show that ET and XGB can classify the flow packets with higher precision. In general, ET achieved the best performance in all metrics, although it had the highest detection or prediction time and memory requirement. Due to the large memory capacity and training and testing time requirement for the ET classifier, we selected XGB and LGBM which had similar performance ratings. On the other hand, DT had a prediction time of 0.18 s. LGBM, being a lightweight model, had the lowest memory requirement of about 3.1 MB with an accuracy of 99.16%. Therefore, the models on both datasets detected and classified each traffic with high performances in comparison with other existing methods [4,12].

We used a stacking method (StackingClassifier) to combine all the algorithms to develop ensembles for each classifier. Hence, we obtained Ens_DT (with DT as meta-learner), Ens_RF (with RF as meta-learner), Ens_LGBM (with LGBM as meta-learner), and Ens_XGB (XGB as meta-learner). To obtain the classifier based on majority vote, (EnsHMV), we used the four base classifiers as estimator and hard voting as the argument for the voting function. BoostedEnML was then developed using LGBM and XGB only.

**Figure 3:**
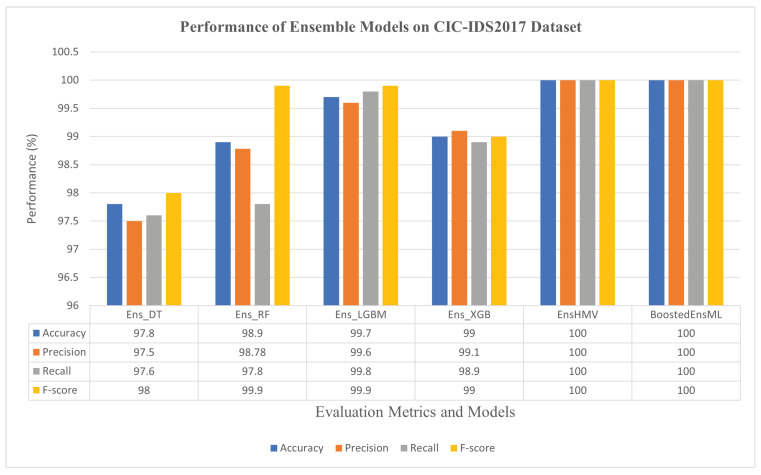
Performance evaluation of ensemble models on the CIC-IDS2017 dataset.

**Figure 4:**
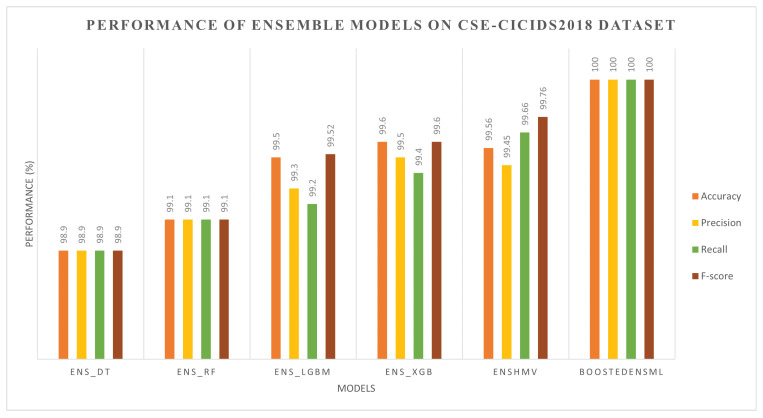
Performance evaluation of ensemble models on the CIC-IDS2018 dataset.

The results obtained for the ensemble approach are shown in Figure 3 and Figure 4, respectively, for the CIC-IDS2017 and CSE-CIC-IDS2018 datasets.

From Figure 3 and Figure 4, we can observe that the ensemble ML classifiers outperformed the single ML classifiers, implying that using the ensemble approach can increase the performance of ML algorithms in detecting cyberattacks in IoT systems. For instance, on the CIC-IDS2017 dataset, Ens_DT, Ens_RF, Ens_LGBM, Ens_XGB, EnsHMV, and BoostedEnsML achieved an accuracy and F1-score of 97.8% and 98%, 98.9% and 99%, 99.7% and 99.9%, 99% and 99%, 99.99% and 99.99%, and 100% and 100%, respectively. The recall and precision in each case lies within the same range. In addition, on the CSE-CIC-IDS2018 dataset, the performance accuracy and recall for each of the ensemble models were, respectively, 98.9% and 98.9%, 99.1% and 99.1%, 99.5% and 99.52%, 99.6% and 99.6%, 99.56% and 99.66%, and 100% and 100% for Ens_DT, Ens_RF, Ens_LGBM, Ens_XGB, EnsHMV, and BoostedEnsML.

**Figure 5:**
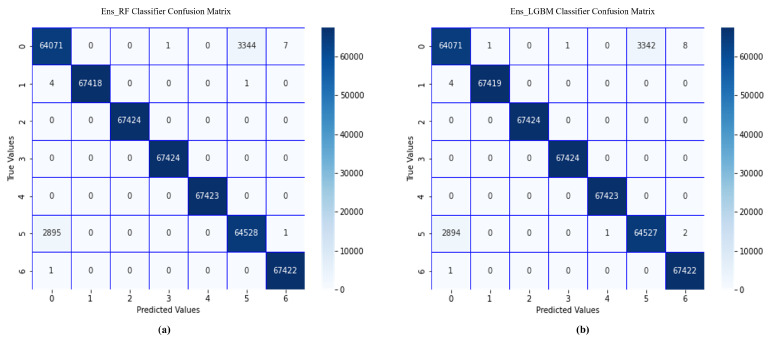
Confusion matrix for (**a**) Ens_RF and (**b**) ENs_LGBM on 2018 dataset.

Since our task is based on multiclass classification, we show the confusion matrix for Ens_RF and Ens_LGBM classifiers in Figure 5. Almost all the various network traffic types were correctly classified. From the confusion matrix, we can see that during the test for brute force, DDoS, and DOS with the Ens_RF model, all the 67,424 data points in the dataset were correctly identified as either brute force, DDoS, or DoS with 100% accuracy. On the other hand, 64,071 instances were identified as benign, 1 instance was misclassified as DDoS, 3344 were misclassified as infiltration attacks, and 7 were misclassified as web attacks while detecting benign traffic on the CSE-CICIDS2018 dataset. Similarly, on the CICIDS2017 dataset, the Ens_LGBM had only 1, 1, 3342, and 8 misclassifications of bot, DDoS, infiltration, and web attacks, respectively, while detecting benign flows, showing an FNR of 0.05%.

**Figure 6:**
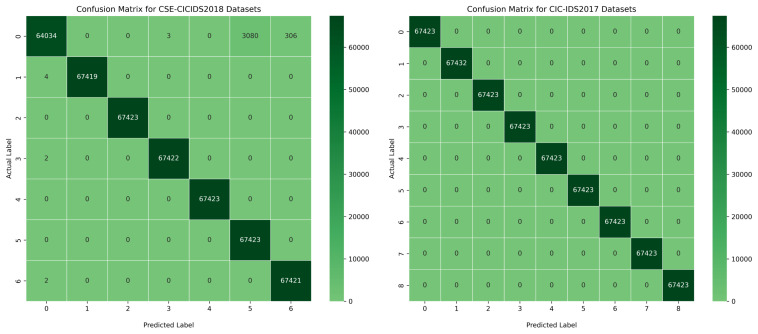
Confusion matrix for EnsHMV.

**Figure 7:**
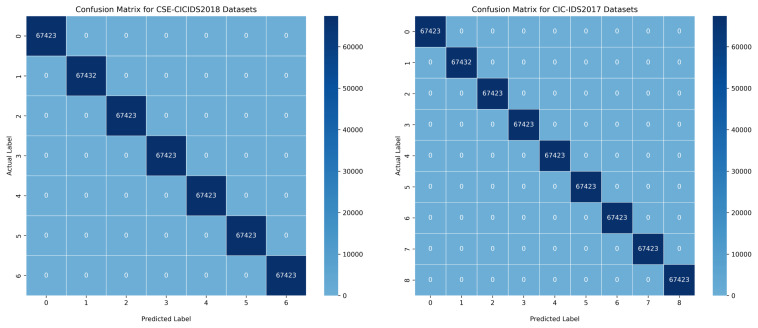
Confusion matix for proposed BoostedEnsML.

However, our proposed BoostedEnsML model outperformed all other ensemble models achieving 100% accuracy, precision, recall, F-score, and AUC for all the different attacks in both datasets, as can be seen in the confusion matrix in Figure 6 and Figure 7. Although other IDS models for IoT scenarios have achieved almost the same accuracy [75], our work demonstrates that using only algorithms based on boosting techniques with balanced datasets can present an improvement on existing works. The model based on the HMV technique, called Ens_HMV, also outperformed other models, reaching high accuracy in both models. Notably, Ens_HMV on the two datasets achieved almost the same performance with the BoostedEnsML model but with regards to memory capacity, the BoostedEnsML (200 MB) is preferred as it has lower computational power than the Ens_HMV (500 MB).

**Figure 8:**
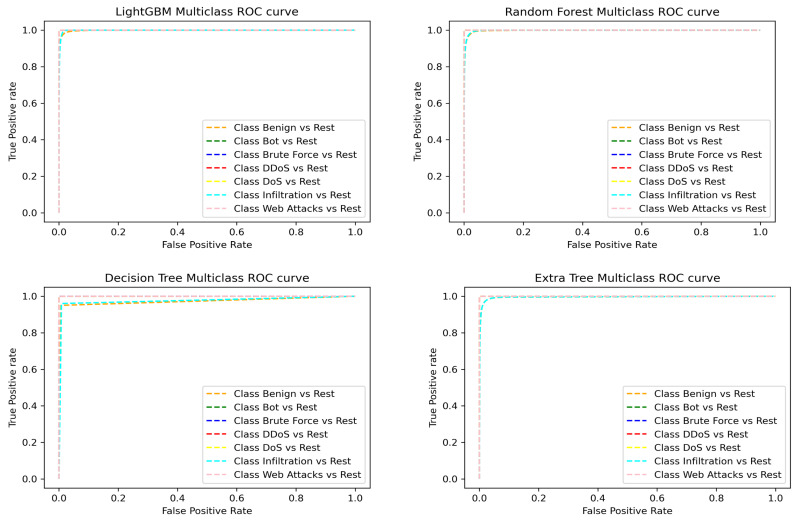
ROC curve for selected models trained on the CSE-CICIDS2018 dataset.

The ROC curve shows the relationship between the true positive rate (TPR) and false positive rate (FPR) for the model performance in detection and classification of each attack. The ROC curve obtained on the CSE-CICIDS2018 dataset for LGBM, RF, DT, and ET is presented in Figure 8. In each case, the AUC score is nearly 1.0, which indicates that the model has high accuracy in correctly classifying the various attacks and benign labels. In addition, the FPR is nearly zero for each of the models, showing a high rate of reduction in false alarms which have been a serious issue in ML used for IDS. Hence, our model outperforms most of the state-of-the-art models [25,26,27] through the methodology adopted for the research. With high detection rate, the proposed model correctly classifies the various network traffic passing through the IoT environment, thereby helping to reduce exposure to cyberattacks.

We applied the ensemble model developed using voting technique (EnsHMV) that is based on bagging classifier for a classification task and the IDS model based on stacked boosting algorithms (BoostedEnML) on each of the datasets to identify how each of them performs in detecting and classifying the network packets into their respective classes. We considered each class as a separate entity to evaluate the classifier’s ability to differentiate it from the normal traffic (benign). The result for this experiment is shown in Table 8. The results illustrate that for the various attack in the two datasets, both IDS models showed high precision, recall, and F-score, reaching 100% in correctly classifying the classes. More specifically, while EnsHMV and BoostedEnML performed similarly on the 2018 dataset, BoostedEnML outperformed EnsHMV on both datasets. In detecting infiltration attacks on the CSE-CICIDS2018 dataset, the two models report that the attack is infiltration with 100% recall, while on CICIDS2017, the EnsHMV detected an infiltration attack with a recall of 99.67% against BoostedEnML that reached 100%. The results generally show a low possibility of false alarms in both scenarios.

In terms of the F-score, which is the weighted mean of the recall and precision of the model behavior, Table 8 demonstrates that the EnsHMV reached 96.36%, 99.84%, 99.99%, 99.89%, 98.90%, 99.69%, 100%, 99.5%, 99.92%, and 99.88% in classifying the benign, botnet, brute force, DDoS, DoS, heartbleed, infiltration, portscan, and web attack traffics in the CICIDS2017 dataset, respectively, while on the CSE-CICIDS2018 dataset, the EnsHMV attained an F-score performance of 99.78%, 100%, 100%, 100%, 99.99%, 99.99%, and 100% in classifying the benign, botnet, brute force, DDoS, DoS, infiltration, and web attack flows, respectively. Similarly, the BoostedEnML showed higher performance than the EnsHMV in relation to the F-score measure on both datasets. Specifically, on the CICIDS2017 dataset, the BoostedEnML showed an F-score of 100% in the classification of the benign, botnet, brute Force, DDoS, Dos, heartbleed, infiltration, portscan, and web attack flows. It also achieved 100% in detecting the benign, botnet, brute force, DDoS, DoS, infiltration, and web attack packets in the CSE-CICIDS2018 dataset.

We compared the performance of our models with those of existing models in the literature, as presented in Table 9. In the work of Das et al. [11], the proposed model achieved an accuracy of 92% for the ensemble decision tree, and our En_DT achieved 97.8%, which is about a 5.8% improvement. In addition, while the ensemble based on the neural network (NN), a deep learning model, achieved 99.5%, our BoostedEnsML achieved 100% in all evaluation metrics, showing that the proposed approach is better. On the same dataset as used in our work, the ensemble model based on stacking RF and KNN with DT used as meta-learner in Kim et al. [75] detected the attacks and benign traffic with the accuracy of 99.9%, while our work detected each traffic with 100%, showing 0.1% improvement after handling data imbalance which was not stated in the work of Kim et al. [75]. This indicates that with a balanced dataset integrated with feature selection, the performance of IDS models can be enhanced. There is also a need to evaluate the effect of different feature selection techniques and data imbalance methods on the general behavior of IDS models in detecting and classifying network flows in IoT systems. Our work will try to investigate this idea in future studies.

## 5. Conclusions

IoT devices are being used in different facets of human endeavors today, leading to the creation of extensive networks and, consequently, a tremendous amount of network data transmission. In addition to this, cyberattacks are witnessed in IoT systems exponentially, prompting the urgency to develop approaches capable of mitigating these attacks. In this paper, we proposed an ensemble model based on boosting algorithms such as XGB and LGBM. First, we solved the data imbalance problem by using two methods of oversampling technique (ADASYN and SMOTE) and compared the results obtained. A balanced dataset obtained with SMOTE showed better performance than that of ADASYN. This case, notwithstanding, can be relative. We performed several experiments on different ML algorithms, including DT, RF, ET, AD, XGB, and LGBM, and developed an ensemble classifier for each model. In the end, the proposed BoostedEnsML model was developed using the best-performing boosting classifiers (XGB and LGBM), achieving the best performance of 100% in the classification of the various attacks in IoT networks, including DDoS, DoS, web attacks, infiltration, portscan, heartbleed, and botnets. BoostedEnsML outperformed all other ensemble models discussed in the literature. Hence, we obtained a highly efficient, reliable, and accurate IDS model for detecting cyberattacks in IoT networks. In this current work, two ensemble models based on boosting techniques (XGB and LGBM) were used to propose an ensemble model using the stacking technique. Future work will explore more ensemble model approaches and deep learning algorithms to further improve IoT intrusion detection. In addition, we hope to integrate more feature selection techniques to evaluate the effects of different network features on the performance of an ensemble IDS model in preventing IoT-based network intrusion, as well as evaluate more boosting algorithms such as Catboost and GBDT, to develop an ensemble of four boosting classifiers.

## Figures and Tables

**Table 1:** Outline of related works that implement machine learning and deep learning in both single and ensemble scenarios for intrusion detection in IoT systems.

Author	Dataset Used	Classification Domain	Imbalance	Method	Evaluation Metric
Rashid et al. [37]	UNSW-NB15, CICIDS2017	Binary	Not specified	Acc = 99.9, Recall = 99.9	Ensemble
Verma et al. [33]	CSE-CICIDS2018-v2, UNSW-NB15-V2, BoT-IoT-V2	Binary	x	Acc = 98.27, Recall = 96.40	Ensemble
Churcher et al. [38]	BoT-IoT	Binary and multiclass	x	Acc = 99	-
Gaikwad and Thool [39]	NSL-KDD	Multiclass	x	Acc = 99.67	Ensemble
Yulianto et al. [55]	CICIDS2017	Multiclass	Implemented	Acc = 81.83, F-score = 90.01	AdaBoost
Waskle et al. [58]	KDD Cup’99	Not specified	x	Acc = 96.78	Random forest
Dhaliwal et al. [62]	NSL-KDD	Multiclass	x	Acc = 98.70, Recall = 99.11	XGBoost
Dutta et al. [74]	IoT-23, LITNET-2020, NetML-2020	Multiclass	Implemented	Acc = 99.7, Precision = 100, Recall = 95	DL Ensemble stacking
Kim et al. [75]	N-BaIoT	Binary and multiclass	x	Acc = 99.9, Recall = 99.9, Precision = 99.9	ML Ensemble stacking
Das et al. [11]	NSL-KDD, UNSW-NB15, CICIDS2017	Binary	x	Acc NSL-KDD: 88.1, UNSW-NB15: 85.7, CICIDS2017: 99.5	Ensemble ML

**Table 2:** Distribution of stream records in CICIDS2017 dataset.

Label Name	Value	Percentage Contribution (%)
BENIGN	2,359,289	83.3452
DoS Hulk	231,073	8.1630
PortScan	158,930	5.6144
DDoS	41,835	1.4779
DoS GoldenEye	10,293	0.3636
FTP-Patator	7938	0.2804
SSH-Patator	5897	0.2083
DoS slowloris	5796	0.2048
DoS Slowhttptest	5499	0.1943
Bot	1966	0.0695
Web Attack-Brute Force	1507	0.0532
Web Attack-XSS	652	0.0230
Infiltration	36	0.0013
Web Attack-Sql Injection	21	0.0007
Heartbleed	11	0.0004

**Table 3:** Distribution of stream records in CICIDS2018 dataset.

Label Name	Value	Percentage Contribution (%)
Benign	13,484,708	83.07001
DDOS attack-HOIC	686,012	4.22605
DDoS attacks-LOIC-HTTP	576,191	3.54952
DoS attacks-Hulk	461,912	2.84552
Bot	286,191	1.76303
FTP-BruteForce	193,360	1.19116
SSH-Bruteforce	187,589	1.15561
Infiltration	161,934	0.99756
DoS attacks-SlowHTTPTest	139,890	0.86177
DoS attacks-GoldenEye	41,508	0.25570
DoS attacks-Slowloris	10,990	0.06770
DDOS attack-LOIC-UDP	1730	0.01066
Brute Force-Web	611	0.00376
Brute Force-XSS	230	0.00142
SQL Injection	87	0.00054

**Table 4:** Selected feature for the training of each of the models using random forest feature importance according to the standard deviation of feature values.

Feature	Importance	Feature	Importance	Feature	Importance	Feature	Importance
Timestamp	0.3227	Fwd IAT Std	0.0022	RST Flag Cnt	0.0009	Pkt Len Min	0.0003
Dst Port	0.2302	Pkt Len Mean	0.0021	Fwd IAT Tot	0.0009	Fwd Pkt Len Min	0.0003
Fwd Seg Size Min	0.16	Tot Bwd Pkts	0.0019	Pkt Size Avg	0.0008	Bwd Seg Size Avg	0.0003
Init Fwd Win Byts	0.0971	Bwd IAT Min	0.0019	Bwd IAT Mean	0.0008	TotLen Bwd Pkts	0.0002
TotLen Fwd Pkts	0.0354	Flow IAT Min	0.0017	Pkt Len Var	0.0007	Idle Min	0.0002
Fwd Pkt Len Mean	0.0347	Init Bwd Win Byts	0.0015	Fwd Header Len	0.0007	Bwd Pkt Len Std	0.0002
Pkt Len Max	0.0334	Fwd Pkts/s	0.0015	Bwd IAT Max	0.0006	Active Min	0.0002
Fwd Pkt Len Std	0.0215	Fwd IAT Max	0.0015	Subflow Bwd Pkts	0.0005	Tot Fwd Pkts	0.0001
Flow IAT Max	0.0057	Flow Pkts/s	0.0015	Pkt Len Std	0.0005	Subflow Fwd Pkts	0.0001
Idle Max	0.0048	Flow IAT Std	0.0015	FIN Flag Cnt	0.0005	PSH Flag Cnt	0.0001
Fwd Pkt Len Max	0.0045	Flow Byts/s	0.0014	Bwd IAT Tot	0.0005	Idle Std	0.0001
Fwd Seg Size Avg	0.0035	Flow IAT Mean	0.0011	Bwd IAT Std	0.0005	Idle Mean	0.0001
Bwd Pkt Len Mean	0.0029	Fwd Act Data Pkts	0.001	Subflow Fwd Byts	0.0004	Active Std	0.0001
Bwd Pkts/s	0.0027	Flow Duration	0.001	Subflow Bwd Byts	0.0004	Active Mean	0.0001
Bwd Pkt Len Max	0.0025	ECE Flag Cnt	0.001	Fwd IAT Mean	0.0004	Active Max	0.0001
Fwd IAT Min	0.0023	Bwd Header Len	0.001	Bwd Pkt Len Min	0.0004	ACK Flag Cnt	0.0001

**Table 5:** Distribution of data for training, validation, and testing of the models.

Labels	CIC-IDS2017		CSE-CICIDS2018	
	Train	Val/Test	Train	Val/Test
**Benign**	606,812	67,242	606,812	67,242
**Bot**	606,812	67,242	606,812	67,242
**Brute force**	606,812	67,242	606,812	67,242
**DDoS**	606,812	67,242	606,812	67,242
**DoS**	606,812	67,242	606,812	67,242
**Infiltration**	606,812	67,242	606,812	67,242
**Web Attacks**	606,812	67,242	606,812	67,242
**Portscan**	606,812	67,242	–	–
**Heartbleed**	606,812	67,242	–	–
**Total**	5,461,308	605,178	4,247,684	470,694

**Table 6:** Performance evaluation of the trained models on CSE-CICIDS2018 dataset, showing the time for prediction and model size.

Model Metrics	Accuracy	Precision	Recall	F-score	AUC	File Size	Test Time (s)
DT	98.7	98.67	98.67	98.67	99.25	10 MB	0.25
RF	98.4	98.43	98.43	98.43	99.93	1200 MB	9.98
ET	98.3	98.35	98.35	98.35	99.85	5500 MB	15.1
AD	97.8	97.74	97.65	97.8	98.8	350 MB	14.2
LGBM	98.8	98.83	98.83	98.83	99.96	**2.4 MB**	3.4
XGB	**98.9**	**98.97**	**98.98**	**98.97**	99.9	1500 MB	4.25

**Table 7:** Performance evaluation of the trained models on the CIC-IDS2017 dataset showing the time for prediction and model size.

Model Metrics	Accuracy	Precision	Recall	F-score	AUC	File Size	Test Time (s)
DT	99.59	99.59	99.59	99.59	99.76	5.7 MB	**0.18**
RF	99.49	99.48	99.47	99.47	99.98	319 MB	6.83
ET	**99.68**	99.68	99.67	99.67	99.97	1630 MB	11.09
AD	69.67	66.79	66.78	66.68	67.9	400 MB	12
LGBM	99.16	96.96	96.43	96.43	96.81	**3.1 MB**	5.49
XGB	**99.51**	**99.52**	**99.51**	**99.51**	99.97	3.76 MB	3.37

**Table 8:** Performance of the IDS models (EnsHMV and BoostedEnsML) in detecting and classifying each network traffic class in the two datasets.

		EnsHMV			BoostedEnML		
**Dataset**	**Class**	**Precision**	**Recall**	**F-Score**	**Precision**	**Recall**	**F-Score**
**CICIDS2017**	Benign	97.95	99.45	96.36	99.89	99.95	100
	Bot	99.77	99.92	99.84	99.97	99.99	99.99
	Brute Force	99.98	99.99	99.99	100	100	99.99
	DDoS	98.80	99.89	98.89	98.90	99.65	99.80
	DoS	99.68	98.90	99.69	100	100	100
	Hearbleed	100	100	100	100	100	100
	Infiltration	99.89	99.67	99.69	100	100	100
	PortScan	99.93	99.92	99.95	99.99	99.99	99.99
	Web Attack	99.66	99.66	99.88	100	100	100
		**Precision**	**Recall**	**F-Score**	**Precision**	**Recall**	**F-Score**
**CICIDS2018**	Benign	99.66	99.90	99.78	99.99	99.99	99.98
	Bot	100	100	100	100	100	100
	Brute Force	99.99	100	100	100	99.99	99.99
	DDoS	99.99	100	100	100	99.99	100
	DoS	99.99	99.99	99.99	99.99	100	99.99
	Infiltration	99.99	100	99.99	100	100	100
	Web Attack	100	100	100	100	100	100

**Table 9:** Comparison of our ensemble models with other state-of-the-art ensembles.

Model Metrics	Accuracy	Precision	Recall	F-score	AUC
**Ens_DT** [11]	92	92	94.4	89.8	96.9
**Ens_SVM** [11]	94	93.5	90.4	97.8	95.3
**Ens_NN** [11]	99.5	99.5	99.6	99.6	99.8
**Ensemble Bagging** [75]	99.7	99.7	99.8	99.8	-
**Ensemble Boosting** [75]	99.8	99.8	99.9	99.9	-
**Ensemble Stacking** [75]	99.9	99.9	99.9	99.9	-
**DNN** [74]	98.4	92	89	87.6	-
**LSTM** [74]	99.1	100	92	95	-
**Ensemble DL Stacking** [74]	99.7	100	95	98	-
**En_DT**	97.8	97.8	97.5	98.0	98.6
**Ens_LGBM**	99.7	99.6	99.8	99.9	99.5
**Ens_XGB**	99.0	99.1	98.9	99.0	99.6
**Ens_HMV**	99.99	100	100	100	99.99
**BoostedEnsML Proposed**	100	100	100	100	100

## Data Availability

The datasets used in this work are publicly available and can be accessed through CIC-IDS2017: http://205.174.165.80/CICDataset/CIC-IDS-2017/Dataset/ and CSE-CICIDS2018: https://registry.opendata.aws/cse-cic-ids2018/ accessed on 2 February 2022.
