# Correction: Okey et al. BoostedEnML: Efficient Technique for Detecting Cyberattacks in IoT Systems Using Boosted Ensemble Machine Learning. *Sensors* 2022, *22*, 7409

**DOI:** 10.3390/s25196125

**Published:** 2025-10-03

**Authors:** Ogobuchi Daniel Okey, Siti Sarah Maidin, Pablo Adasme, Renata Lopes Rosa, Muhammad Saadi, Dick Carrillo Melgarejo, Demóstenes Zegarra Rodríguez

**Affiliations:** 1 Department of Systems Engineering and Automation, Federal University of Lavras, Lavras 37203-202, MG, Brazil; 2 Faculty of Data Science and Information Technology (FDSIT), INTI International University, Nilai 71800, Malaysia; 3 Department of Electrical Engineering, University of Santiago de Chile, Santiago 9170124, Chile; 4 Department of Computer Science, Federal University of Lavras, Lavras 37200-000, MG, Brazil; 5 Department of Electrical Engineering, University of Central Punjab, Lahore 54000, Pakistan; 6 Department of Electrical Engineering, School of Energy Systems, Lappeenranta-Lahti University of Technology, FI-53851 Lappeenranta, Finland

## Text Correction

There was an error in the original publication [1]. A correction has been made due to an error in the presentation of the percentage values in paragraph 13 in the Section 4 Results and Discussion.

In terms of the F-score, which is the weighted mean of the recall and precision of the model behavior, Table 8 demonstrates that the EnsHMV reached 96.36%, 99.84%, 99.99%, 99.89%, 98.90%, 99.69%, 100%, 99.5%, 99.92%, and 99.88% in classifying the benign, botnet, brute force, DDoS, DoS, heartbleed, infiltration, portscan, and web attack traffics in the CICIDS2017 dataset, respectively, while on the CSE-CICIDS2018 dataset, the EnsHMV attained an F-score performance of 99.78%, 100%, 100%, 100%, 99.99%, 99.99%, and 100% in classifying the benign, botnet, brute force, DDoS, DoS, infiltration, and web attack flows, respectively. Similarly, the BoostedEnML showed higher performance than the EnsHMV in relation to the F-score measure on both datasets. Specifically, on the CICIDS2017 dataset, the BoostedEnML showed an F-score of 100% in the classification of the benign, botnet, brute Force, DDoS, Dos, heartbleed, infiltration, portscan, and web attack flows. It also achieved 100% in detecting the benign, botnet, brute force, DDoS, DoS, infiltration, and web attack packets in the CSE-CICIDS2018 dataset.

## Error in Table

In the original publication, there was a mistake in the data in Table 8. The correct data appears below.

The authors state that the scientific conclusions are unaffected. This correction was approved by the Academic Editor. The original publication has also been updated.

**Table 8:** Performance of the IDS models (EnsHMV and BoostedEnsML) in detecting and classifying each network traffic class in the two datasets.

		EnsHMV			BoostedEnML		
**Dataset**	**Class**	**Precision**	**Recall**	**F-Score**	**Precision**	**Recall**	**F-Score**
**CICIDS2017**	Benign	97.95	99.45	96.36	99.89	99.95	100
	Bot	99.77	99.92	99.84	99.97	99.99	99.99
	Brute Force	99.98	99.99	99.99	100	100	99.99
	DDoS	98.80	99.89	98.89	98.90	99.65	99.80
	DoS	99.68	98.90	99.69	100	100	100
	Hearbleed	100	100	100	100	100	100
	Infiltration	99.89	99.67	99.69	100	100	100
	PortScan	99.93	99.92	99.95	99.99	99.99	99.99
	Web Attack	99.66	99.66	99.88	100	100	100
		**Precision**	**Recall**	**F-Score**	**Precision**	**Recall**	**F-Score**
**CICIDS2018**	Benign	99.66	99.90	99.78	99.99	99.99	99.98
	Bot	100	100	100	100	100	100
	Brute Force	99.99	100	100	100	99.99	99.99
	DDoS	99.99	100	100	100	99.99	100
	DoS	99.99	99.99	99.99	99.99	100	99.99
	Infiltration	99.99	100	99.99	100	100	100
	Web Attack	100	100	100	100	100	100
